# Early prolonged neutrophil activation in critically ill patients with sepsis

**DOI:** 10.1177/1753425920980078

**Published:** 2021-01-18

**Authors:** Sanna Törnblom, Sara Nisula, Suvi T Vaara, Meri Poukkanen, Sture Andersson, Ville Pettilä, Eero Pesonen

**Affiliations:** 1 Division of Intensive Care Medicine, Department of Anaesthesiology, Intensive Care and Pain Medicine, University of Helsinki and Helsinki University Hospital, Finland; 2 Department of Anaesthesia and Intensive Care Medicine, Lapland Central Hospital, Finland; 3 New Children’s Hospital, University of Helsinki and Helsinki University Hospital, Finland; 4 Division of Anaesthesiology, Department of Anaesthesiology, Intensive Care and Pain Medicine, University of Helsinki and Helsinki University Hospital, Finland

**Keywords:** Heparin binding protein, interleukin 6, interleukin 8, myeloperoxidase, sepsis

## Abstract

We hypothesised that plasma concentrations of biomarkers of neutrophil activation and pro-inflammatory cytokines differ according to the phase of rapidly evolving sepsis. In an observational study, we measured heparin-binding protein (HBP), myeloperoxidase (MPO), IL-6 and IL-8 in 167 sepsis patients on intensive care unit admission. We prospectively used the emergence of the first sepsis-associated organ dysfunction (OD) as a surrogate for the sepsis phase. Fifty-five patients (of 167, 33%) developed the first OD > 1 h before, 74 (44%) within ± 1 h, and 38 (23%) > 1 h after intensive care unit admission. HBP and MPO were elevated at a median of 12 h before the first OD, remained high up to 24 h, and were not associated with sepsis phase. IL-6 and IL-8 rose and declined rapidly close to OD emergence. Elevation of neutrophil activation markers HBP and MPO was an early event in the evolution of sepsis, lasting beyond the subsidence of the pro-inflammatory cytokine reaction. Thus, as sepsis biomarkers, HBP and MPO were not as prone as IL-6 and IL-8 to the effect of sample timing.

## Introduction

Incidence of sepsis, a life-threatening organ dysfunction (OD) caused by a dys-regulated host response to infection,^1^ is increasing.^2^,^3^ Current evidence suggests that sepsis-associated OD, such as acute kidney injury (AKI), is predominantly associated with inflammation, not ischaemia/hypotension of the organ.^4^ Prompt recognition of sepsis, leading to earlier administration of antibiotics and start of supportive therapies, has improved survival.^5^ However, interventions targeting inflammation have failed.^6^

Sepsis develops when the immune system fails to restrict the infection at the local site. The outbreak of bacteraemia leads to activation of TLRs and systemic inflammation.^7^ In addition, circulating neutrophils are activated.^8^ In experimental sepsis, systemic activation of neutrophils results in their impaired migration to the site of infection due to down-regulated surface expression of the chemokine receptor CXCR2. Up-regulation of CC receptor expression, however, mediates infiltration of neutrophils in the lungs, kidneys and heart.^7^ These changes in expression of neutrophil chemokine receptors have been observed in septic patients.^7^ Taken together, systemic neutrophil activation is harmful for the septic patient by two means: it reduces clearance of bacteria at the site of infection and paradoxically increases the risk of neutrophil-mediated OD.^7^,^8^

Heparin-binding protein (HBP) is a 37-kD granule protein of neutrophils.^9^ As a sensitive indicator of intravascular neutrophil activation, HBP has gained increasing interest as a promising inflammatory biomarker in severe sepsis and septic shock.^10^ Among septic patients, it predicts development of circulatory failure,^11^ respiratory failure,^12^ AKI^13^,^14^ and OD in general.^15^ Myelo-peroxidase (MPO), a traditional neutrophil activation marker, is released from the azurophilic granules of neutrophils.^16^ Plasma MPO concentrations have been shown to correlate inversely with neutrophil CXCR2 expression.^17^ We have recently shown the association of urinary MPO with septic AKI.^18^ IL-6 is a pleiotropic cytokine possessing both pro- and anti-inflammatory properties,^19^ whereas IL-8 is a neutrophil chemotactic factor,^20^,^21^ which primes neutrophils in septic patients.^22^ Both these pro-inflammatory cytokines have been associated with AKI,^23–25^ disease severity and mortality.^23–26^

Investigation of the kinetics of the inflammatory reaction in sepsis is difficult because patients typically present with intense inflammation on hospital admission. Here we studied the kinetics of systemic neutrophil activation (HBP and MPO) and pro-inflammatory reaction (IL-6 and IL-8) in early sepsis in critically ill patients, using the emergence of the first sepsis-associated OD as a surrogate for the sepsis phase. We hypothesised that baseline plasma levels and kinetics of IL-6, IL-8, HBP and MPO differ depending on the phase of sepsis.

## Materials and methods

### Patients

**Figure 1.**
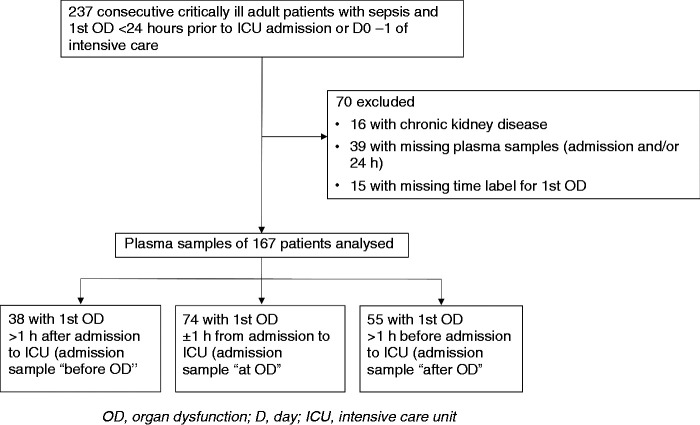
Flow chart of the study cohort.

We investigated the kinetics of plasma HBP, MPO, IL-6 and IL-8 in the early phase of sepsis in a previously described subgroup of adult patients^18^ originating from the prospective nationwide FINNAKI study.^27^ Plasma concentrations of MPO, IL-6 and IL-8 have been previously related to development of AKI.^18^ We screened 237 consecutive sepsis patients (Flow chart in Figure 1) manifesting their first OD between 24 h preceding admission and the second calendar day in the intensive care unit (ICU) (that is, by 48 h following admission). Sepsis was defined according to the American College of Chest Physicians/Society of Critical Care Medicine (ACCP/SCCM) criteria.^28^ To be included in the study, the patient had to have an infection or a strong suspicion of infection, systemic inflammatory response syndrome, and at least one OD due to infection. This definition corresponds closely to the later Sepsis 3.0 criteria for sepsis.^1^ The screened OD comprised acute respiratory, cardiovascular, acute kidney and liver dysfunctions, hypotension despite adequate fluid resuscitation, metabolic acidosis, disseminated intravascular coagulation, and hypoperfusion (lactatemia, oliguria or decreased level of consciousness). We excluded patients with chronic kidney disease (glomerular filtration rate less than 60 ml/min/1.73 m^2^), those without both admission and 24 h plasma samples available, and those without a recorded time label for the first sepsis-associated OD. The final number of included patients was 167 (Figure 1, Table 1). In addition, we assayed HBP, MPO, IL-6 and IL-8 in 20 healthy adult controls.

**Table 1.** Baseline characteristics of critically ill sepsis patients (*n* = 167).

			Data available
Age, median [IQR]		62 [53 − 72]	167
Male gender, *n* (%)		108 (65)	167
Comorbidities, *n* (%)	Hypertension	77 (47)	165
	Insulin or oral medication for diabetes	34 (20)	167
	Atherosclerosis	17 (10)	165
	Systolic heart failure	12 (7)	165
Admission type and status	Emergency, *n* (%)	164 (98)	167
	Operative, *n* (%)	36 (22)	166
	SAPS II, median [IQR]	43 [35−52]	167
	APACHE II, median [IQR]	23 [19−29]	167
Severity of disease and organ dysfunction	Septic shock in first 5 ICU days, *n* (%)	118 (71)	167
	Lactate, mmol/l (first in ICU, median [IQR])	1.6 [1.0−3.5]	134
	SOFA < 24 h, median [IQR]	8 [6−11]	167
	Highest SOFA, median [IQR]	10 [7−12]	167
	AKI, *n* (%)	85 (51)	167
	RRT for AKI, *n* (%)	23 (14)	167
ICU LOS, days, median [IQR]		4.5 [2.7−8.1]	167

Data expressed as median [IQR] or count (%). IQR: interquartile range; SAPS: Simplified Acute Physiology Score; APACHE: Acute Physiology and Chronic Health Evaluation; SOFA: Sequential Organ Failure Assessment; AKI: acute kidney injury; RRT: renal replacement therapy; ICU: intensive care unit; LOS: length of stay.

The local ethics committee approved the FINNAKI study (18/13/03/02/2010), from which the study cohort was derived, with use of a deferred consent, as well as recruitment of the control cohort (HUS/606/2020). We obtained a written informed consent from each patient or his/her proxy and from each volunteer of the control cohort.

### Clinical data and onset of sepsis

We prospectively collected clinical data using a standardised case report form (CRF) filled on admission and daily thereafter by the responsible clinician. The CRF data included screening for sepsis and sepsis-associated OD 24 h preceding admission, on ICU admission day, and each day thereafter until discharge or d 5 in the ICU. The clinicians documented the starting date and time (hh:mm) for each emerging OD when any of the following pre-defined criteria were met:

- Hypotension: systolic arterial pressure (SAP) ≥90 mmHg, or reduction of over 40 mmHg from baseline, or inotrope/vasopressor treatment.
- Hypoperfusion: lactate above the reference value, or oliguria, or acutely altered mental status.
- Acute respiratory dysfunction: PaO_2_/FiO_2_ ratio <200 mm Hg, if respiratory dysfunction only, and <250 mm Hg, if other ODs present.
- Acute kidney dysfunction: urine output ≤0.5 ml/kg for one h despite adequate fluid resuscitation.
- Acute cardiovascular dysfunction: SAP ≥ 90 mm Hg or mean arterial pressure (MAP) ≤70 mmHg for at least an h.
- Acute haematological dysfunction: thrombocytes ≤80 E9/l or ≥50% decrease in 3 d.
- Unexplained metabolic acidosis: pH ≤7.30, or base excess (BE) ≤–5 and lactate >1.5 times normal likely to be caused by infection.

We collected physiological data including Acute Physiology and Chronic Health Evaluation II (APACHE II), Simplified Acute Physiology Score II (SAPS II), and Sequential Organ Failure Assessment (SOFA) scores of the first 5 d of ICU stay from the quality database of the Finnish Intensive Care Consortium maintained by Tieto Ltd, Helsinki, Finland.

### Blood samples

Blood samples were obtained in ethylenediaminetetraacetic acid tubes on ICU admission. In the subgroup with the first OD close to admission (sample ‘at OD’, see Statistical analysis section), blood samples taken 24 h after admission were also analysed. The tubes were centrifuged and aliquoted immediately after ICU admission or at 2 h at the latest and stored at –80°C until assayed. Including aliquoting and analyses, the samples were thawed a maximum of three times. Control samples were collected in the same way. To analyse both study and control samples we used commercial ELISA kits for IL-6 (DiaClone, Besancon, France; detection limit 2 pg/ml), IL-8 (Cymax, Abfrontier, Seoul, South-Korea; detection limit 0.3 pg/ml), HBP (Axis-Shiled Diagnostics, Dundee, UK; detection limit 5.9 ng/ml) and MPO (BioLegend Inc., San Diego, CA, USA; detection limit 28 pg/ml) analyses. The researchers conducting the analyses were blinded to the subgrouping (see Statistical analysis section) of the study patients.

### Statistical analysis

We divided the patients into three groups based on the time of the first OD in relation to ICU admission. We used cut-off points of 1 h before and after admission. Because blood samples were taken at the time of ICU admission, the groups were named according to the time of blood sampling in relation to OD: sample ‘before OD’ (the first OD over 1 h after admission), ‘at OD’ (the first OD ± 1 h from admission) and ‘after OD’ (the first OD over 1 h before admission; Table 2). As relevant publications for calculation of statistical power did not exist, we used the results of an unpublished pilot cohort of 32 sepsis patients to conduct the power analysis for the study. Because sample size cannot be calculated for a non-parametric test, we based sample size estimation on a one-way ANOVA, which is equivalent to the Kruskal–Wallis test. To gain the sample size for a non-parametric test, we added 10% to the number of patients, as suggested. The eta-square for IL-6 was 0.316 and for IL-8 0.360. The minimum group size for the one-way ANOVA was 33 patients for IL-6 and 26 patients for IL-8 (α = 0.05, 1-β = 0.80). Consequently, the minimum patient number for Kruskal–Wallis test would have been 37 patients. For HBP and MPO, power analysis was not applicable because there were no differences in their levels between the time points in the pilot cohort.

**Table 2.** Characteristics of the three groups with different sampling time in relation to emergence of organ dysfunction.

	Data available	Before OD (*n* = 38)	At OD (*n* = 74)	After OD (*n* = 55)	*p*
Time from ICU admission to first documented OD, h, median (range)	167	12.3 (1.0−45.6)	0 (−0.9−0.9)^a^	−4.1 (−23.8−−1.2)^a^	
Age, median [IQR]	167	60 [50−71]	63 [56−73]	61 [52−72]	0.310
Male gender, *n* (%)	167	24 (63)	46 (62)	38 (69)	0.700
Comorbidities, *n* (%)					
Hypertension	165	15 (40)	31 (42)	31 (56)	0.238
Diabetes with oral or insulin medication	167	8 (21)	12 (16)	14 (26)	0.433
Atherosclerosis	165	4 (11)	7 (10)	6 (11)	0.853
Systolic heart failure	165	3 (8)	7 (10)	2 (4)	0.451
Admission type and status					
Emergency, *n* (%)	167	38 (100)	72 (97)	54 (98)	0.797
Operative, *n* (%)	166	13 (34)	6 (8)	17 (31)	0.001
SAPS II, median [IQR]	167	41 [32−51]	44 [35−56]	44 [36−50]	0.459
APACHE II, median [IQR]	167	23 [18−29]	24 [19−30]	23 [20−29]	0.510
Sepsis severity and organ dysfunction					
Septic shock (first 5 ICU days, *n* (%)	167	26 (68)	49 (66)	43 (78)	0.317
Lactate, mmol/l (first in ICU, median [IQR])	134	1.5 [1.1−2.1]	1.6 [1.0−3.7]	1.6 [1.0−4.0]	0.612
Highest SOFA, median [IQR]	167	9 [8−12]	9 [7−11]	10 [7−12]	0.342
Respiratory SOFA ≥2, *n* (%)	167	34 (90)	65 (88)	53 (96)	0.208
CNS SOFA ≥2, *n* (%)	153	19 (50)	37/70 (53)	21/45 (47)	0.810
Coagulation SOFA ≥2, *n* (%)	167	13 (34)	21 (28)	19 (35)	0.707
Liver SOFA ≥2, *n* (%)	158	9/36 (25)	11/70 (16)	12/52 (23)	0.438
AKI, *n* (%)	167	19 (50)	34 (46)	32 (58)	0.386
RRT for AKI, *n* (%)	167	4 (11)	10 (14)	9 (16)	0.722
ICU LOS, d, median [IQR]	167	4.6 [2.9−8.3]	4.1 [2.6−8.0]	5.0 [2.7−8.2]	0.856

‘Before OD’: patients who developed their first organ dysfunction > 1 h after ICU admission; ‘At OD’: patients presenting their first organ dysfunction ± 1 h from admission; ‘After OD’: patients who had organ dysfunction > 1 h before ICU admission. ^a^A negative value indicates that organ dysfunction has emerged before admission. ICU: intensive care unit; IQR: interquartile range; SAPS: Simplified Acute Physiology Score; APACHE: Acute Physiology and Chronic Health Evaluation; SOFA: Sequential Organ Failure Assessment; CNS: central nervous system; AKI: acute kidney injury; RRT: renal replacement therapy; LOS: length of stay.

As HBP, MPO, IL-6 and IL-8 plasma concentrations were not normally distributed, non-parametric tests (Friedman, Kruskal–Wallis, Mann–Whitney and Wilcoxon tests) were used, as appropriate. Proportions were compared with chi-square or Fisher’s exact test and bivariate correlations with the non-parametric Spearman test. A pairwise comparison was used as a post hoc test for the Kruskal–Wallis test. A *P* value < 0.05 was considered significant. The data are expressed as numbers and percentages, as median with interquartile range (IQR) or range (data involving time), or depicted as box plots with median, IQR and range. Statistical analyses were conducted using SPSS 22 software (SPSS Inc., Chicago, IL, USA).

## Results

The baseline characteristics of the patients (*n* = 167) are presented in Table 1. The median time between ICU admission and the emergence of the first OD was 0.0 h, ranging from 23.8 h before admission to 45.6 h after admission. Of 167 patients, 38 (23%) developed their first OD later than 1 h after admission (blood sampling ‘before OD’), 74 (44%) within an interval of ± 1 h from admission (blood sampling ‘at OD’), and 55 (33%) presented with the first documented OD earlier than 1 h before admission (blood sampling ‘after OD’; Figure 1, Table 2). The baseline characteristics of these three groups are shown in Table 2. The groups were comparable regarding comorbidities, disease severity and presence of different ODs (Table 2).

**Figure 2.**
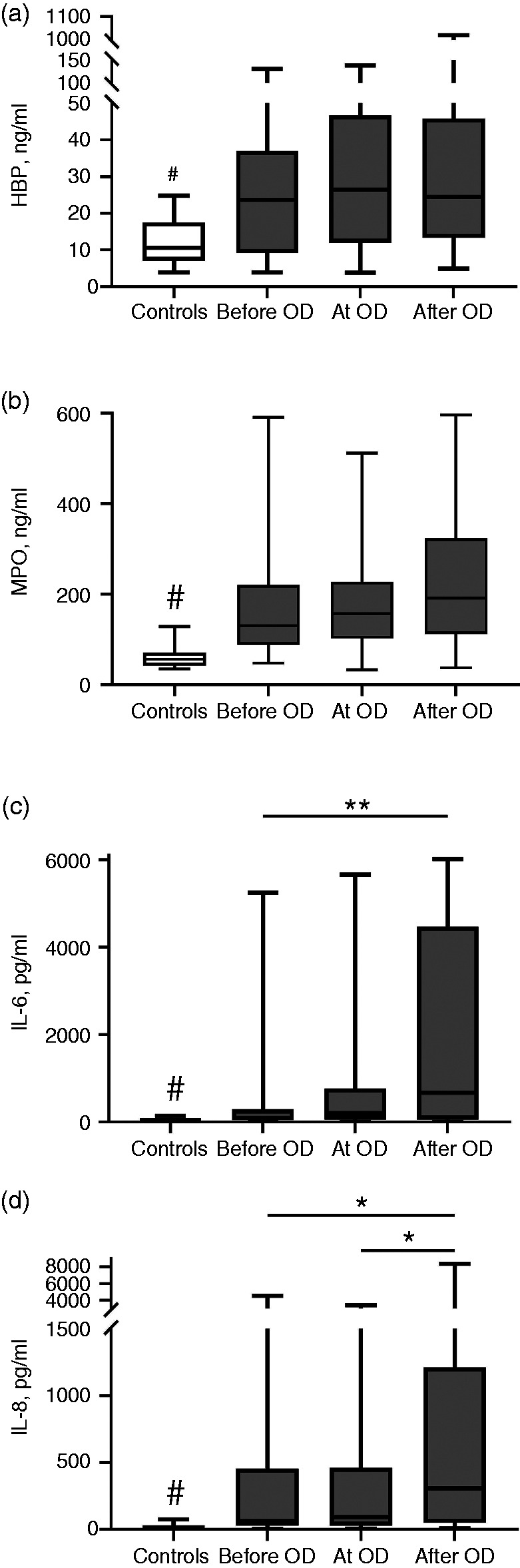
Inflammatory biomarkers a) HBP, b) MPO, c) IL-6, and d) IL-8 in controls and in the three groups with different sampling time in relation to the onset of organ dysfunction (‘before OD’, ‘at OD’ and ‘after OD’). ^#^*P* < 0.01, controls vs. before OD/at OD/after OD; *P < 0.05, among sepsis patients; **P < 0.01, among sepsis patients. a) HBP b) MPO c) IL-6 d) IL-8.

**Figure 3.**
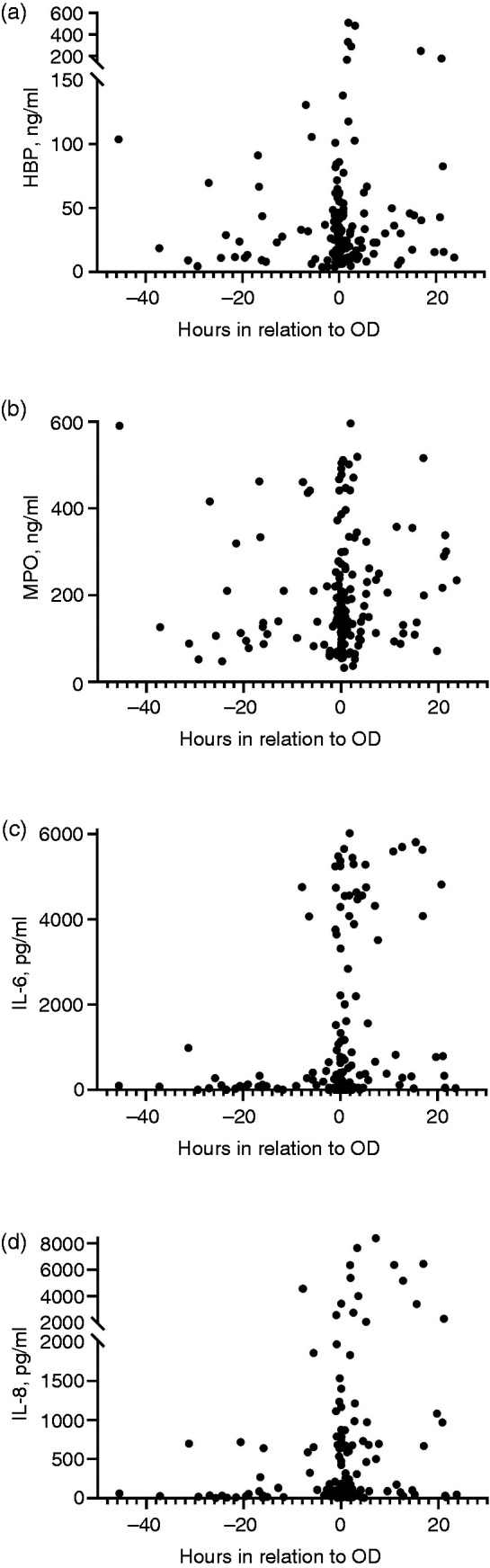
Concentrations of inflammatory biomarkers a) HBP, b) MPO, c) IL-6, and d) IL-8 in relation to emergence of organ dysfunction (OD). Negative values on x-axis indicate h before first OD and positive values indicate h after first OD.

We analysed IL-6 and MPO of all 167 patients. Due to an insufficient amount of plasma, we could not analyse IL-8 of two patients and HBP of four patients. All four biomarkers of the 20 healthy controls with median age 34 [3142] yr, 8 (40%) males and 12 (60%) females were analysed. Among 167 sepsis patients, there was a strong positive correlation between HBP and MPO (*r* = 0.737, *P* < 0.001) as well as between IL-6 and IL-8 (*r* = 0.811, *P* < 0.001) on admission. Positive correlations were also observed between neutrophil activation markers (MPO and HBP) and IL-6 and -8 with correlation coefficients ranging from 0.309 to 0.477 (*P* < 0.001 for all). The concentrations of the biomarkers in controls and in the three patient groups with different onset of OD compared with blood sampling time are presented in Figure 2. Healthy controls had lower concentrations than sepsis patients in all studied biomarkers (Figure 2). Plasma HBP or MPO concentrations did not differ between the three groups, but levels of IL-6 (*P* < 0.01) and IL-8 (*P* < 0.05) increased close to the onset of the first OD (Figures 2 and 3). Figure 3 illustrates the concentrations of the biomarkers relative to OD.

**Figure 4.**
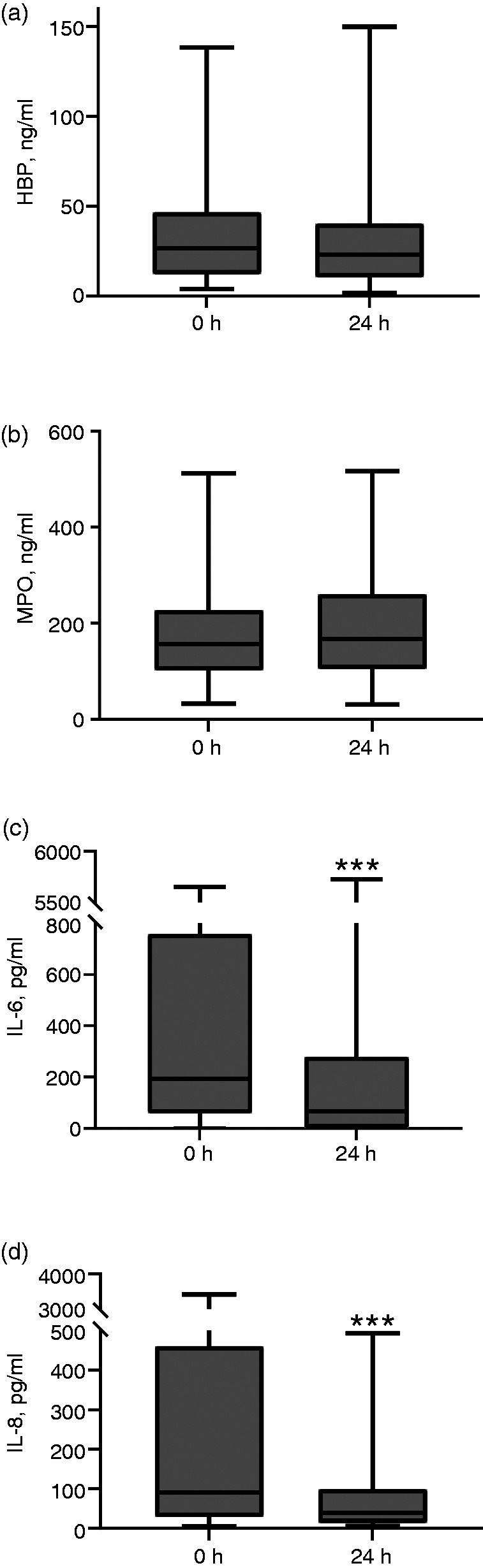
Inflammatory biomarkers a) HBP, b) MPO, c) IL-6, and d) IL-8 in the subgroup of patients with onset of organ dysfunction ± 1 h from ICU admission at 0 h (admission) and 24 h later (24 h; *n* = 74). ***P < 0.001

Among patients with the first OD ± 1 h from admission (subgroup with sample ‘at OD’, *n* = 74), we compared the plasma concentrations measured 24 h after ICU admission with the concentrations on admission (Figure 4). A statistically significant decline between 0 and 24 h was observed in IL-6 and IL-8, but not in HBP or MPO.

## Discussion

In this study comprising adult critically ill septic patients we found that intravascular neutrophil activation measured by plasma HBP and MPO concentrations had steadily elevated before the onset of sepsis and remained elevated up to 24 h. Unexpectedly, systemic neutrophil activation did not follow the kinetics of pro-inflammatory cytokines: the rise and fall of plasma IL-6 and IL-8 concentrations followed rapid kinetics close to the onset of the first OD.

### Neutrophil activation

In this study, prospectively documented emergence of the first sepsis-associated OD was used as a surrogate for the sepsis phase. In the subgroup ‘before OD’, median plasma HBP was 23.7 ng/ml and median plasma MPO 130.2 ng/ml at a median of 12 h before the first OD, whereas healthy controls had a median HBP concentration of 10.6 ng/ml and MPO concentration of 56 ng/ml. In other words, plasma concentrations of neutrophil activation biomarkers HBP and MPO were markedly increased early in the course of sepsis. In agreement, increased plasma HBP concentrations have been detected in sepsis up to 12 h before circulatory failure and up to 24 h before OD.^11^,^15^ Furthermore, neutrophil activation was not limited to the emergence of sepsis. Corroborating findings in HBP,^10^ neutrophil activation (by HBP and MPO) lasted at least for the first 24 h following the emergence of the first OD.

### Interplay between neutrophil activation and pro-inflammatory cytokines

The kinetics of the pro-inflammatory cytokines differed distinctly from the neutrophil activation markers. The present results suggest that the increase of IL-6 and IL-8 occurs within hours. The rise of the pro-inflammatory cytokines was sharp around the onset of the first OD. In experimental endotoxemia in healthy volunteers, IL-6 and IL-8 concentrations increased steeply within 1 to 2 h, resembling our findings.^29^,^30^ In a murine model of caecal ligation and puncture, IL-6 and IL- 8 increased more steadily and peaked at 16 h.^31^ We detected higher plasma IL-6 and IL-8 concentrations in the subgroup of patients presenting with the first OD median of 4 h before ICU admission than in patients with onset of OD on ICU admission. However, the latter patients showed lower pro-inflammatory cytokine concentrations at 24 h after ICU admission than at the time of admission. These results suggest that the peak of IL-6 and IL-8 occurred at some point during the first 24 h after evolution of the first OD. The decline of the pro-inflammatory cytokines seems to occur rapidly as well. Assessed among the subgroup with sampling ‘at OD’, the pro-inflammatory cytokines decreased profoundly during the first 24 h following the first OD. Our results are consistent with three previous clinical studies in severe sepsis.^23^,^25^,^32^

Pro-inflammatory cytokines probably do not have cytotoxic effects by themselves. Instead, they are mediators and regulators of pathophysiological processes. IL-8 is an important endogenous chemotactic factor of neutrophils^20^ and primes neutrophils in septic patients.^22^ Nevertheless, according to the present results, circulating IL-8 does not seem to be linked to intravascular neutrophil activation in septic patients. It should be noted, however, that we measured inflammation at the systemic level. Indeed, local cytokine concentrations at the site of infection and at organ level may be different. Furthermore, it has been suggested that microbiological agents may directly activate neutrophils resulting in elevated plasma HBP concentrations.^33^

### Clinical implications

As for the clinical implications of the present results there are two aspects to consider. First, neutrophil activation can be detected for hours before emergence of OD. Although it is practically impossible to modify excessive neutrophil activation in a safe way, the consequences of it might be alleviated. As an example, heparin reduces HBP-mediated endothelial hyperpermeability in experimental conditions.^34^ If this kind of intervention has clinical significance, such treatment should be started as early as possible on patient admission to the hospital rather than waiting until ICU admission.

Second, from the biomarker point of view, HBP and MPO remained stably elevated between consecutive time points. In contrast, a difference of only a few hours in sample timing may have a significant effect in the obtained IL-6 and IL-8 concentrations. Obviously, neutrophil activation markers are not as prone as pro-inflammatory cytokines to the effect of sample timing, and HBP has been suggested to be superior to IL-6 as a biomarker of sepsis.^11^ In a recent study, sepsis phenotypes were distinguished by inflammatory biomarkers, which were highest among the phenotypes with the highest mortality.^35^ As an early and relatively stable inflammatory biomarker, HBP may help to recognise high-risk sepsis patients.

### Limitations and strengths

Our study has some limitations and strengths. First, a key question is whether the post hoc grouping of the patients according to the emergence of the first OD in relation to admission and sampling carries confounding factors influencing the interpretation of the results. This does not seem to be the case as the three groups were comparable in terms of comorbidities as well as ODs and disease severity. Second, the recorded time for the first OD is not always accurate in patients who develop OD before ICU admission. OD, although present, might not be monitored and diagnosed until in the ICU. Third, we did not study neutrophil function directly (e.g. phagocytosis, oxidative burst or receptor expression). Fourth, at the enrollment of the study cohorts the new Sepsis 3.0 definition did not exist.^1^ However, we checked post hoc whether the patients fulfilled the Sepsis 3.0 criteria. Only one patient had a SOFA score change < 2 during the first 24 h in ICU. Therefore, we used the term sepsis instead of severe sepsis. The main strength of our study is the prospective documentation of the onset of the first OD. Furthermore, we used two parallel biomarkers for detection of both neutrophil activation (HBP and MPO) and pro-inflammatory reaction (IL-6 and IL-8) to confirm our findings.

### Conclusion

We found that systemic neutrophil activation, measured by HBP and MPO, occurs early and lasts for at least 24 h in the evolution of sepsis. This contrasts the rapid changes in IL-6 and IL-8 concentrations close to the onset of the first OD. Implications of ongoing neutrophil activation on sepsis pathophysiology and performance of neutrophil granule proteins as sepsis biomarkers need to be further addressed.
